# TIPS Placement via Combined Transjugular and Transhepatic Approach for Cavernous Portal Vein Occlusion: Targeted Approach

**DOI:** 10.1155/2013/635391

**Published:** 2013-01-21

**Authors:** Natanel Jourabchi, Justin Pryce McWilliams, Edward Wolfgang Lee, Steven Sauk, Stephen Thomas Kee

**Affiliations:** ^1^Department of Interventional Radiology, David Geffen School of Medicine, UCLA Health System, Los Angeles, CA 90095, USA; ^2^Mallinckrodt Institute of Radiology, Washington University, Campus Box 8131, 510 South Kingshighway Boulevard, St. Louis, MO 63110, USA

## Abstract

*Purpose*. We report a novel technique which aided recanalization of an occluded portal vein for transjugular intrahepatic portosystemic shunt (TIPS) creation in a patient with symptomatic portal vein thrombosis with cavernous transformation. Some have previously considered cavernous transformation a contraindication to TIPS. *Case Presentation*. 62-year-old man with chronic pancreatitis, portal vein thrombosis, portal hypertension and recurrent variceal bleeding presents with melena and hematemesis. The patient was severely anemic, hemodynamically unstable, and required emergent portal decompression. Attempts to recanalize the main portal vein using traditional transjugular access were unsuccessful. After percutaneous transhepatic right portal vein access and navigation of a wire through the occluded main portal vein, an angioplasty balloon was inflated at the desired site of shunt takeoff. The balloon was targeted and punctured from the transjugular approach, and a wire was passed into the portal system. TIPS placement then proceeded routinely. *Conclusion*. Although occlusion of the portal vein increases difficulty of performing TIPS, it should not be considered an absolute contraindication. We have described a method for recanalizing an occluded portal vein using a combined transhepatic and transjugular approach for TIPS. This approach may be useful to relieve portal hypertension in patients who fail endoscopic and/or surgical therapies.

## 1. Introduction 

Transjugular intrahepatic portosystemic shunt (TIPS) is a well-established treatment for the complications of portal hypertension. There are few absolute contraindications for TIPS; right-sided heart failure, severe liver failure, and polycystic liver disease are most commonly cited [1, 2]. Portal vein thrombosis increases the technical difficulty of the TIPS procedure, but TIPS is still often desirable due to the high likelihood of variceal bleeding and ascites in these patients [3–5]. 

As portal vein thrombosis becomes more chronic, the occluded portal vein becomes fibrotic and shrunken, and cavernous transformation occurs, with collateral formation in the hepatic hilum. The occluded portal vein becomes difficult to access by the transjugular route, and even if the portal vein or one of the collaterals is accessed, wire passage across the occluded segment may be difficult or impossible [6]. Some authors have considered cavernous transformation to be a contraindication to TIPS placement [2, 3, 6, 7]. In this report, we describe a novel technique which may increase the technical success of the TIPS procedure in these patients.

## 2. Case Report 

**Figure 1:**
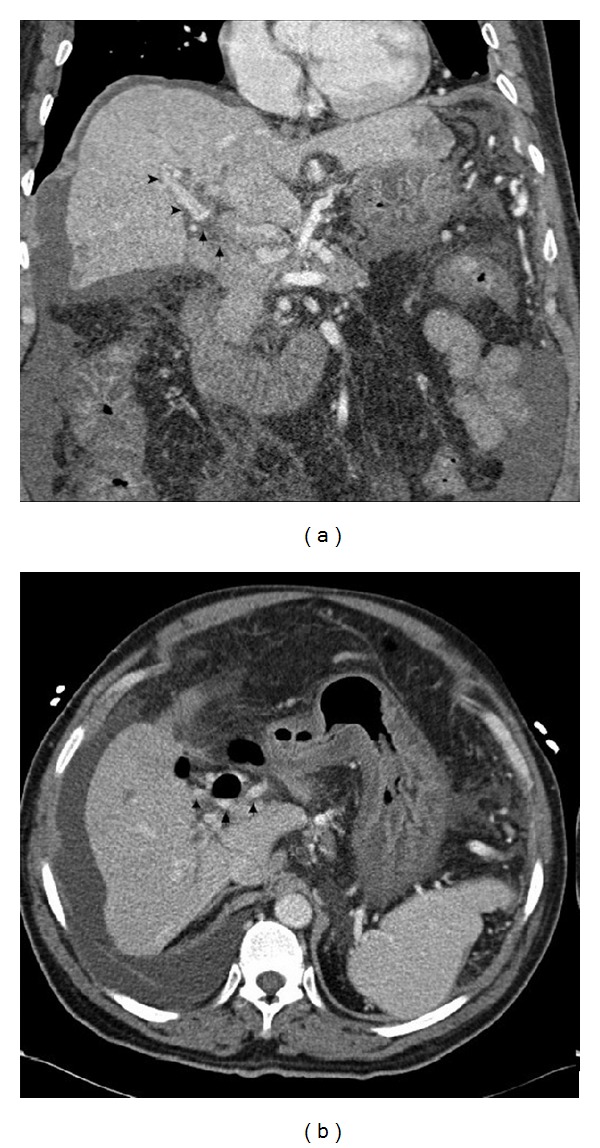
62-year-old man with portal vein thrombosis with cavernous transformation. Preprocedural coronal CT image demonstrates complete occlusion of the main portal vein (arrows), with reconstitution of intrahepatic branches (arrowheads) (a). Preprocedural axial CT image better demonstrates the multiple cavernous collaterals at the level of the hepatic hilum (arrows) (b).

Institutional review board (IRB) approval was obtained for this case presentation. A 62-year-old man with a history of chronic pancreatitis, resultant portal vein thrombosis, and portal hypertension with recurrent variceal bleeding presented to the emergency room with several bouts of melanotic stool and one episode of large volume hematemesis. His blood pressure was 81/50, heart rate was 105, and hemoglobin was 6.0 (baseline 9.9). Aggressive volume resuscitation was instituted, and emergent endoscopy demonstrated large gastric varices which were not amenable to endoscopic therapy. CT demonstrated portal vein occlusion with cavernous transformation (Figure 1). A decompressive mesocaval shunt was attempted by surgery, but was unsuccessful. At surgery, diffuse plastic peritonitis was present throughout the upper abdomen, likely as a result of chronic pancreatitis. The vascular structures could not be isolated. Excessive bleeding occurred during the operation, requiring significant blood product replacement, and following stabilization, the procedure was terminated. The patient was returned to the surgical intensive care unit (ICU), and the decision was made to refer the patient to interventional radiology to try a TIPS placement as a “last ditch effort” for portal decompression. 

 At the time of procedure, the patient was intubated and sedated, but hemodynamically stable. The right internal jugular vein was accessed, and a 10-French Rosch-Uchida system (Cook Inc., Bloomington, IN) was placed. The right hepatic vein was cannulated, and the sheath was advanced. A balloon-occlusion CO2 portogram was performed, demonstrating patent intrahepatic portal vein branches, but complete occlusion of the main portal vein.

**Figure 2:**
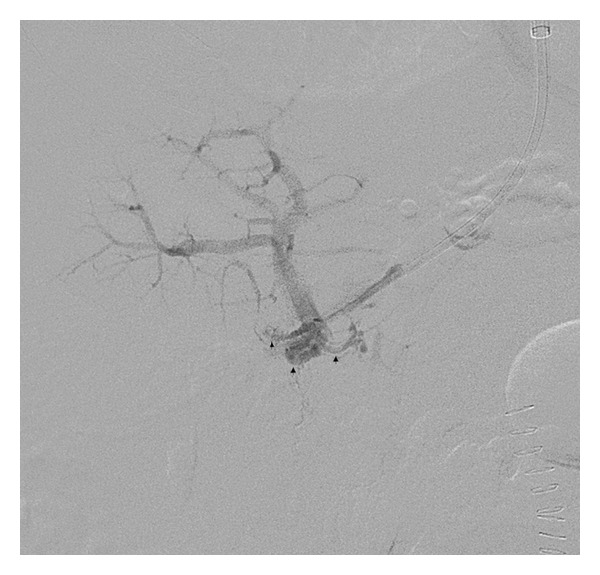
62-year-old man with portal vein thrombosis with cavernous transformation. Angiogram following transjugular access to the portal system demonstrates access into a network of cavernous collaterals (arrows), with patent intrahepatic branches. The wire could not be navigated through the occluded main portal vein from this approach.

Under fluoroscopic guidance, the Rosch-Uchida system needle was used to puncture into the portal venous system. Contrast injection demonstrated multiple portal venous collaterals consistent with cavernous transformation (Figure 2). Multiple attempts to traverse the occluded portal vein using an angled Glidewire were unsuccessful. The Rosch-Uchida needle was withdrawn, and the decision was made to attempt a combined transjugular-transhepatic approach.

**Figure 3:**
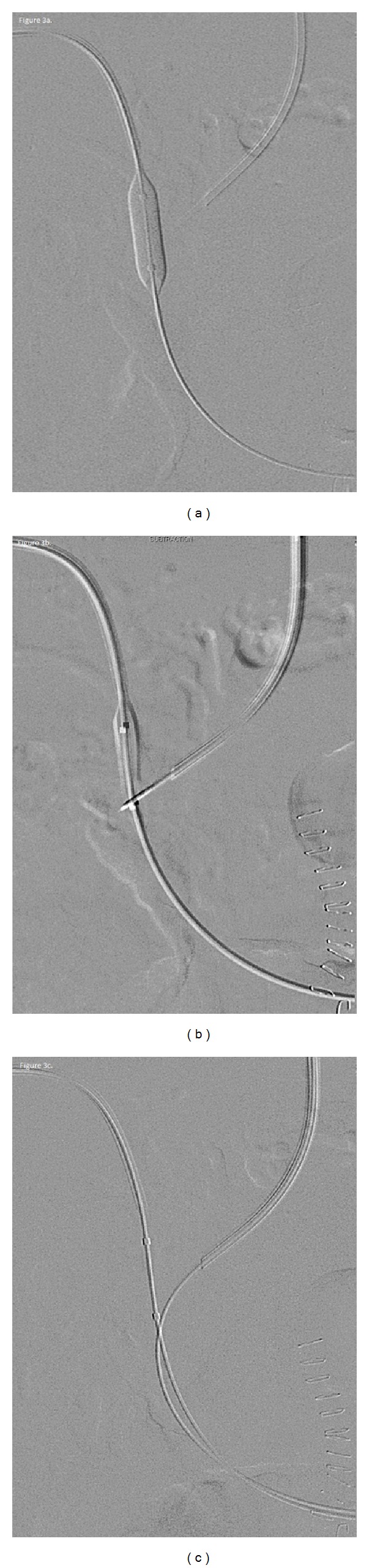
62-year-old man with portal vein thrombosis with cavernous transformation. Sequential digital subtraction images following traversal of the main portal vein occlusion from the transhepatic approach. An angioplasty balloon is inflated within the occluded main portal vein (a). The Rosch-Uchida needle from the transjugular approach is used to puncture the balloon (b). An angled Glidewire is passed through the Rosch-Uchida needle into the portal system (c).

Under ultrasound guidance, a 22-gauge Chiba needle (Cook, Inc., Bloomington, IN) was advanced from the right mid-axillary line into a right portal vein branch, and a Neff set (Cook, Inc., Bloomington, IN) was placed. A 4-French angled glide catheter and 0.035′′ angled Glidewire (Terumo, Somerset, NJ) were advanced to the level of portal vein occlusion, and with repetitive advancement and spinning of the glidewire, the occlusion was traversed. A 6-French vascular sheath was placed. Next, an 8 mm × 2 cm Optapro balloon (Cordis Corporation, Miami, FL) was positioned at the site of desired portal vein access and inflated with contrast (Figure 3(a)). Under fluoroscopic guidance, with the contrast-filled balloon as a target, the transjugular Rosch-Uchida needle was used to puncture the balloon (Figure 3(b)). Following balloon rupture, the glidewire advanced easily through the needle into the portal system (Figure 3(c)).

**Figure 4:**
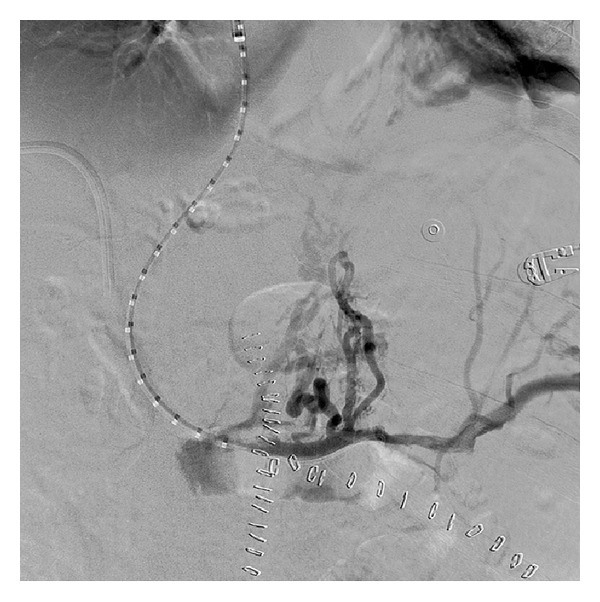
62-year-old man with portal vein thrombosis with cavernous transformation. Portography from the splenic vein demonstrates filling of multiple varices and nonfilling of the occluded portal vein. The portosystemic gradient is 23 mm Hg.

**Figure 5:**
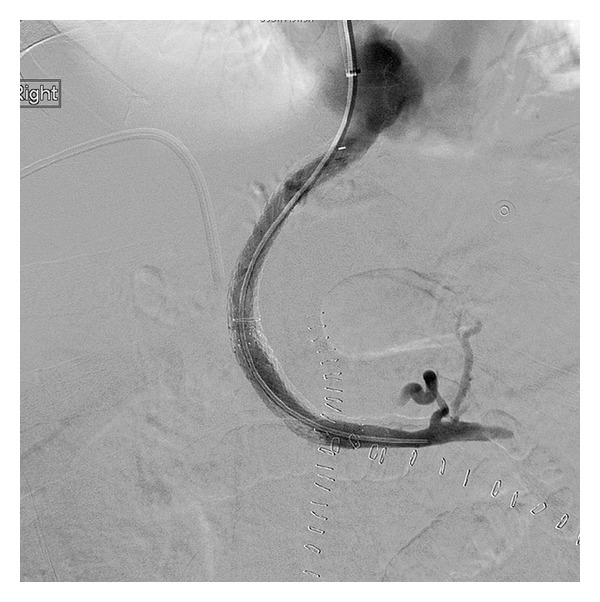
62-year-old man with portal vein thrombosis with cavernous transformation. Rapid inline flow through the TIPS following stent-graft placement from the main portal vein to the proximal right hepatic vein and bare metal stent placement in the more proximal main portal vein. The final portosystemic gradient is 11 mm Hg.

A 5-French pigtail catheter (Cook Inc., Bloomington, IN) was placed, and pressure measurements were obtained, demonstrating a portosystemic gradient of 23 mm Hg. Portography demonstrated chronic occlusion of the portal vein at the confluence of the superior mesenteric vein and splenic vein, with multiple varices (Figure 4). Over a 0.035′′ Amplatz wire (Cook Inc, Bloomington, IN), angioplasty of the portal-hepatic vein tract was performed with 6 mm × 4 cm and 8 mm × 4 cm balloons. The 10-French transjugular sheath was then manipulated into the portal vein, and a 10 mm × 8 cm × 2 cm Viatorr stent-graft (Gore, Flagstaff, AZ) was deployed from the portal vein to the hepatic vein/IVC confluence. The stent-graft was dilated using a 10 mm × 4 cm balloon. Repeat pressure measurements demonstrated persistence of a significant portosystemic gradient, and angiography demonstrated an area of stenosis proximal to the stent-graft, near the splenic vein-SMV confluence. This was treated with a 10 mm × 6 cm Protégé bare metal stent (eV3, Plymouth, MN). Final portography demonstrated excellent flow through the TIPS without significant variceal filling (Figure 5). The final portosystemic gradient was 11 mm Hg. The transhepatic sheath was removed with gelfoam embolization of the parenchymal tract.

The patient's postprocedure course was uncomplicated. He was extubated, his diet was advanced, and he was discharged home in stable condition one week after the TIPS procedure. One year after the TIPS, the patient is well, without further complications related to portal hypertension. The TIPS remains widely patent.

## 3. Discussion 

TIPS is effective in the management of portal hypertensive complications, including bleeding gastroesophageal varices and refractory ascites [8]. Thrombotic occlusion of the portal vein increases the difficulty of the procedure and has prompted several variations in technique to improve technical success [7, 9–11]. However, when the thrombosis is chronic and associated with cavernous transformation, the success of TIPS has remained limited. In such patients, Jiang et al. reported a 20% (1/5) success rate, with less symptomatic improvement and shorter survival time than noncavernous patients [7]. Walser et al. failed in both patients they attempted (0/2) [6]. Senzolo et al. reported a 67% (6/9) success rate, and Van Ha et al. reported a 75% (3/4) success rate [5]. Two isolated case reports have described success [12, 13].

There are several challenges in performing TIPS in cavernous portal vein occlusion. Transjugular access to the portal system may result in puncture of a cavernous collateral, as occurred in our patient, rather than a normal intrahepatic branch. This limits the ability to navigate a wire through the occluded main portal vein. Also, some of these collateral vessels are low pressure and will not provide adequate portal decompression [14]. Percutaneous transhepatic right portal vein access as we have described, in contrast, allows the recanalization to initiate from a normal, patent intrahepatic portal vein, thereby favoring passage into the main portal vein. It also provides more secure portal access, a more direct angle of approach to the occlusion, and allows usage of various wire and catheter combinations to cross the occluded segment. 

Once the portal vein is recanalized via the transhepatic approach, the remaining step is to connect the portal and systemic circulations. A previously described approach, the “gun-sight” approach, directs intravascular snares with a separate transhepatic puncture [15]. We found it far simpler to inflate an angioplasty balloon at the site of desired portal vein access; this simultaneously dilated the occluded portal vein, creating a channel, while also serving as a target for the needle from the transjugular approach. Passage of the needle into the channel is marked by balloon rupture, following which the wire can be easily advanced. TIPS placement then proceeds routinely. 

## 4. Conclusion

Although cavernous occlusion of the portal vein increases the difficulty of performing TIPS, it should not be considered an absolute contraindication. We have described a method for percutaneous recanalization of an occluded portal vein and combined transhepatic and transjugular approach for TIPS creation in a patient with symptomatic chronic portal vein thrombosis associated with cavernous transformation. This approach may be useful to relieve portal hypertension in patients who fail endoscopic and/or surgical therapies.

## Abbreviations

TIPS: Transjugular intrahepatic portosystemic shunt
ICU: Intensive care unit.
